# Multiomics Screening Identified CpG Sites and Genes That Mediate the Impact of Exposure to Environmental Chemicals on Cardiometabolic Traits

**DOI:** 10.3390/epigenomes8030029

**Published:** 2024-07-29

**Authors:** Majid Nikpay

**Affiliations:** Omics and Biomedical Analysis Core Facility, University of Ottawa Heart Institute, Ottawa, ON K1Y 4W7, Canada; mnikpay@ottawaheart.ca or majid.nikpay@gmail.com; Tel.: +1-613-722-3532

**Keywords:** environmental chemicals, epigenome, cardiometabolic traits, exposome, Mendelian randomization, multiomics

## Abstract

An understanding of the molecular mechanism whereby an environmental chemical causes a disease is important for the purposes of future applications. In this study, a multiomics workflow was designed to combine several publicly available datasets in order to identify CpG sites and genes that mediate the impact of exposure to environmental chemicals on cardiometabolic traits. Organophosphate and prenatal lead exposure were previously reported to change methylation level at the cg23627948 site. The outcome of the analyses conducted in this study revealed that, as the cg23627948 site becomes methylated, the expression of the *GNA12* gene decreases, which leads to a higher body fat percentage. Prenatal perfluorooctane sulfonate exposure was reported to increase the methylation level at the cg21153102 site. Findings of this study revealed that higher methylation at this site contributes to higher diastolic blood pressure by changing the expression of *CHP1* and *GCHFR* genes. Moreover, *HKR1* mediates the impact of B12 supplementation → cg05280698 hypermethylation on higher kidney function, while *CTDNEP1* mediates the impact of air pollution → cg03186999 hypomethylation on higher systolic blood pressure. This study investigates CpG sites and genes that mediate the impact of environmental chemicals on cardiometabolic traits. Furthermore, the multiomics approach described in this study provides a convenient workflow with which to investigate the impact of an environmental factor on the body’s biomarkers, and, consequently, on health conditions, using publicly available data.

## 1. Introduction

With the advances in technology, exposure of the human body to various chemicals occurs often and through different paths such as ingestion, inhalation, skin contact and via the umbilical cord to the unborn child. According to a World Health Organization report [1], over one third (35%) of the cases of ischemic heart disease, the leading cause of deaths and disability worldwide, and about 42% of strokes, the second largest contributor to global mortality, could be prevented by reducing or removing exposure to chemicals. The United States Center for Disease Control and Prevention reported the presence of 148 different environmental chemicals in samples of blood and urine taken from the US population [2]; however, despite such issues, the benefits of synthetic chemicals to everyday life are undeniable; humans will continue to synthesize new chemicals that did not previously exist [3]. Therefore, from the clinical perspective, research on the impact of exposure to environmental chemicals is needed for the purposes of risk assessment, early diagnosis, and therapeutic interventions.

As reviewed earlier [4,5], in addition to altering the sequence of DNA, the epigenome is also considered a path through which an environmental chemical can alter the transcriptome and cause a disease. The epigenome is a molecular interface that records the interactions between external factors and the body in the form of chemical modifications. These modifications consequently provide information for the transcriptome machinery to adjust the expressions of genes in order to maintain the homeostasis of the body in response to external stimuli such as exposure to an environmental chemical.

Over the past decades, numerous studies have catalogued the effects of environmental chemicals on epigenomes; information from such studies has been recorded in publicly available databases. In this field of research, several challenges still exist, including questions concerning the epigenomic consequences of exposure to an environmental chemical on disease risk as well as the underlying molecular mechanisms. In the past, answering such questions required extensive experiments and longitudinal studies; however, with the availability of high-throughput screening methods, there is an alternative solution to investigate such questions computationally.

High-throughput screening studies have generated data for various biological features such as epigenomes, transcriptomes, phenomes, etc. Currently, there are initiatives to connect these data through multiomics studies for downstream applications. A variant of these studies uses the genome (i.e., SNPs) as a central axis with which to connect various sources of molecular data and evaluate the nature of the relations between them [6,7]. This is a notable paradigm, because over the past two decades, genome-wide association studies (GWAS) have been able to quantify the impact of SNPs on various biological features; computational tools have been developed that can process these data to find significant relations between biological features. This addresses an important limitation in current epidemiological studies because, to investigate the relation between two phenotypes in an epidemiological study, it is important to collect data on the same group of participants; however, this is not always feasible. Current developments in the field of GWAS provide the means with which to investigate the relations between phenotypes obtained from different groups of participants.

In line with these developments, this study describes a workflow that, by combining publicly available datasets, aims to investigate the molecular path through which an environmental chemical causes a disease. The technical details and the source of the data used in this study are explained in the Materials and Methods Section. In the Results Section, the utility of the approach is described by reviewing the outcomes of analyses.

## 2. Results

**Figure 1:**
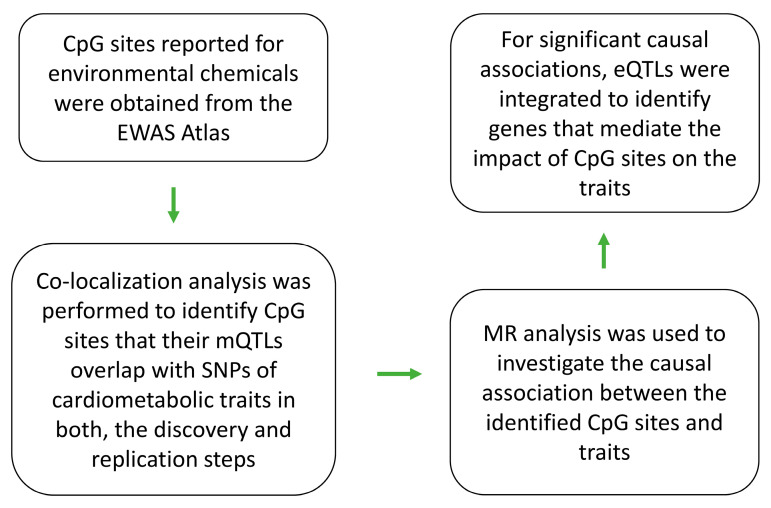
Overview of the multiomics approach used in this study to investigate the molecular path through which an environmental chemical impacts a cardiometabolic trait. Initially, the list of CpG sites that underwent chemical modification as a result of exposure to environmental chemicals were obtained from the EWAS Atlas. Then, colocalization analysis was performed to identify genomic regions that the SNPs underlying a CpG site and a cardiometabolic trait correlate with. Significant CpG–trait pairs from this stage were then subjected to Mendelian randomization to determine if changes in the methylation level at a CpG site have a causal impact on a cardiometabolic trait (*p* < 5 × 10^−8^). Finally, to obtain functional insight, eQTL data from the eQTLGen consortium were integrated to investigate genes that convey the impact of a CpG site on a trait.

By following the analysis pipeline described in Figure 1, four CpG sites were identified in which their mQTLs colocalized with GWAS signals for cardiometabolic traits in both the discovery (Table 1) and replication steps (Table 2). The list of environmental chemicals associated with these sites, as ascertained in the EWAS atlas, are provided in Table 3. The findings from the colocalization analysis were further confirmed by Mendelian randomization (MR) (Table 4). The outcome of the forward MR analysis revealed that changes in the methylation level at the identified CpG sites have causal impacts on their corresponding traits. In addition, the reverse MR ruled out (*p* > 0.05) the possibility of reverse causation. Then, eQTL summary association statistics from the eQTLGen consortium were integrated into the analyses to investigate genes that convey the impact of methylation sites on the traits (Table 4). In the following sections, the findings are discussed in detail by focusing on each CpG site.

### 2.1. cg23627948-GNA12-Obesity

**Figure 2:**
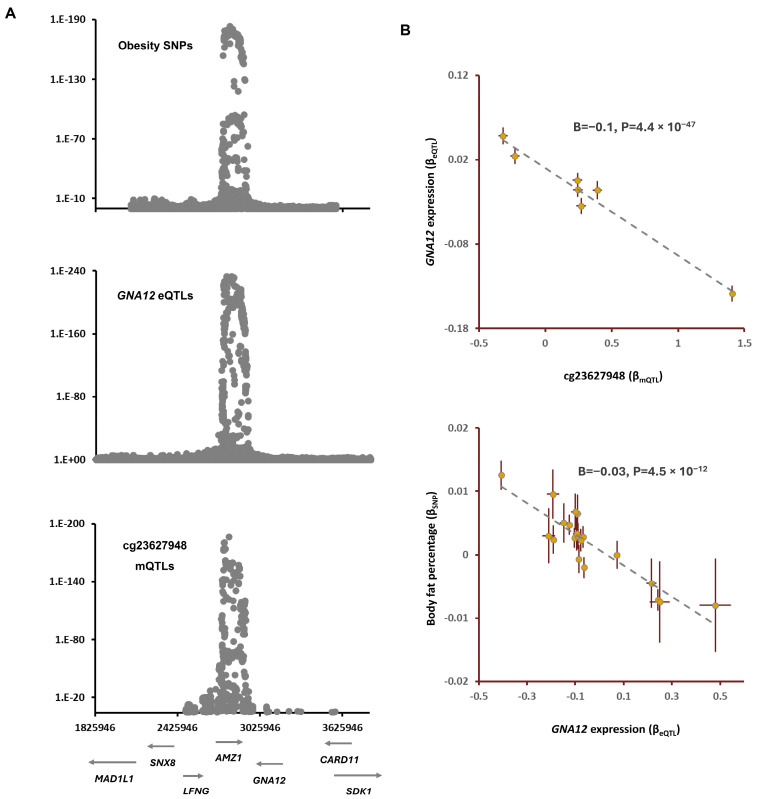
The mechanism whereby cg23627948 mediates the impact of environmental chemicals on obesity: (**A**) regional association plots for mQTLs of cg23627948, eQTLs of *GNA12*, and risk SNPs of obesity overlap; and (**B**) the cg23627948 site is reported to undergo chemical modification as a result of exposure to environmental factors such as organophosphates and lead (Table 3). Findings from the MR analysis indicated that higher methylation at cg23627948 leads to lower expression of *GNA12*; this consequently contributes to higher body fat percentage. Complete statistical details are available in Table 4. Points on MR plots represent SNPs; the x-value of an SNP is its effect size on the predictor, the horizontal error bar indicates the standard error around the effect size. Similarly, the y-value of the SNP indicates its effect size on the outcome, and the vertical error bar indicates the standard error. The dashed line represents the line of best fit (a line with the intercept of 0 and the slope of B from the MR test).

Previous studies documented the impact of environmental factors, organophosphate [8] and prenatal lead exposure [9], on the degree of methylation at the CpG site cg23627948 within the chromosome band 7p22 (Table 3). Colocalization analysis revealed that the mQTLs of cg23627948 overlap (P_SMR_ = 1.4 × 10^−8^, P_HEIDI_ = 0.04, Table 1) with risk SNPs for body fat percentage (BFP). The lead SNP, rs798549-C in this region, was associated with higher methylation at the cg23627948 site (B = 1.4, *p* < 1 × 10^−200^) and higher BFP (B = 0.01, *p* = 1.3 × 10^−8^, Table 1). The outcome of the replication analysis confirmed this finding (Table 2). The MR analysis revealed that higher methylation at the cg23627948 site contributes to higher BFP (B = 0.01, *p* = 1.0 × 10^−8^, Table 4). By integrating eQTL data, I noted that eQTLs of *GNA12* overlap with the GWAS signal for BFP and mQTLs of cg05228408 (Figure 2). The outcome of the MR analysis indicated that, as the methylation at the cg23627948 site increases, the expression of *GNA12* decreases (B = −0.1, *p* = 4.4 × 10^−47^); this leads to higher BFP (B = −0.03, *p* = 4.5 × 10^−12^, Figure 2). *GNA12* encodes a subunit of the guanine-nucleotide-binding protein known as G12-protein alpha subunit.

### 2.2. cg21153102-CHP1/GCHFR-DBP

**Figure 3:**
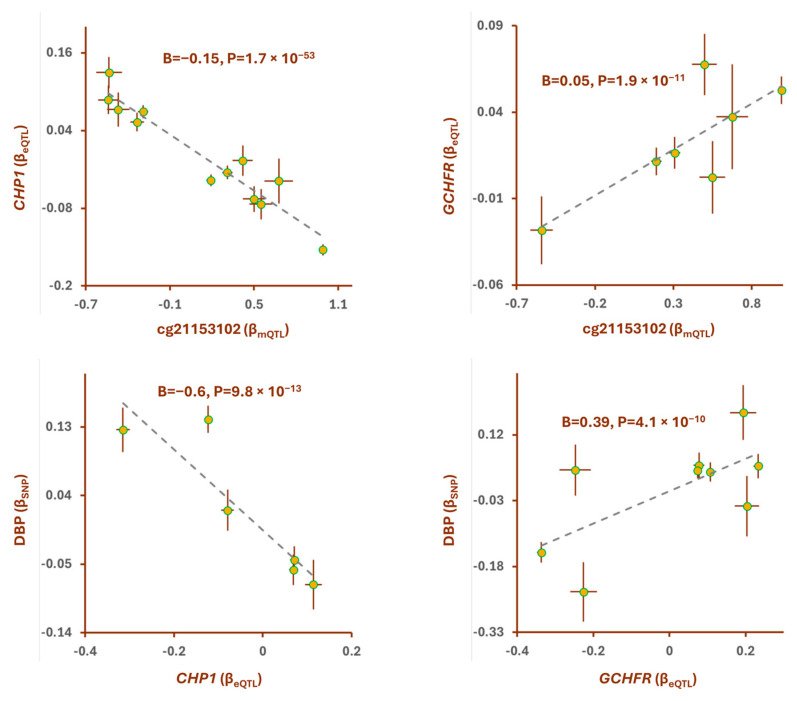
Higher methylation at cg21153102 site contributes to diastolic blood pressure (DBP) by changing the expression of *GCHFR* and *CHP1*. cg21153102 undergoes chemical modification as a result of exposure to perfluorooctane sulfonate (Table 3). I noted that as the cg21153102 site becomes methylated it increases the expression of *GCHFR* but lowers levels of *CHP1*. This consequently leads to higher DBP, because a higher expression of *GCHFR* and a lower level of *CHP1* are associated with higher DBP levels. Complete statistical details are available in Table 4. Points on MR plots represent SNPs; the x-value of an SNP is its effect size on the predictor, and the horizontal error bar indicates the standard error around the effect size. Similarly, the y-value of the SNP indicates its effect size on the outcome, and the vertical error bar indicates the standard error. The dashed line represents the line of best fit (a line with the intercept of 0 and the slope of B from the MR test).

Within chromosome 15q15.1, I found the methylation site, cg21153102, that becomes methylated due to prenatal perfluorooctane sulfonate exposure (Table 3). The outcome of the SMR analysis indicated that the mQTLs of cg21153102 and the SNPs for diastolic blood pressure (DBP) colocalize (P_SMR_ = 8.0 × 10^−22^, P_HEIDI_ = 0.3; Table 1). The lead SNP in this locus, rs4924526(A), was associated with higher methylation at the cg21153102 site (B = 1, *p* < 1 × 10^−200^) and a higher risk of DBP (B = 0.2, *p* = 3 × 10^−23^; Table 1). The outcome of the MR analysis confirmed that higher methylation at the cg21153102 site is causally associated with higher DBP (B = 0.2, *p* = 1.8 × 10^−23^; Table 4). By integrating the eQTL data, I found that genes *CHP1* and *GCHFR* mediate the impact of methylation at the cg21153102 site on DBP (Figure 3). Higher methylation at the cg21153102 site was associated with lower expression of *CHP1* (B = −0.15, *p* = 1.7 × 10^−53^) but higher expression of *GCHFR* (B = 0.05, *p* = 1.9 × 10^−11^). Further analyses revealed that the higher expression of *CHP1* contributes to lower DBP (B = −0.6, *p* = 9.8 × 10^−13^, Figure 3), whereas *GCHFR* expression has the opposite effect.

*CHP1* encodes a phosphoprotein that acts as an endogenous inhibitor of calcineurin activity and also serves as an essential cofactor for the activity of the sodium–hydrogen antiporter gene family. *GCHFR* encodes an enzyme that is involved in the biosynthesis of tetrahydrobiopterin.

### 2.3. cg05280698-HKR1-Kidney Function

**Figure 4:**
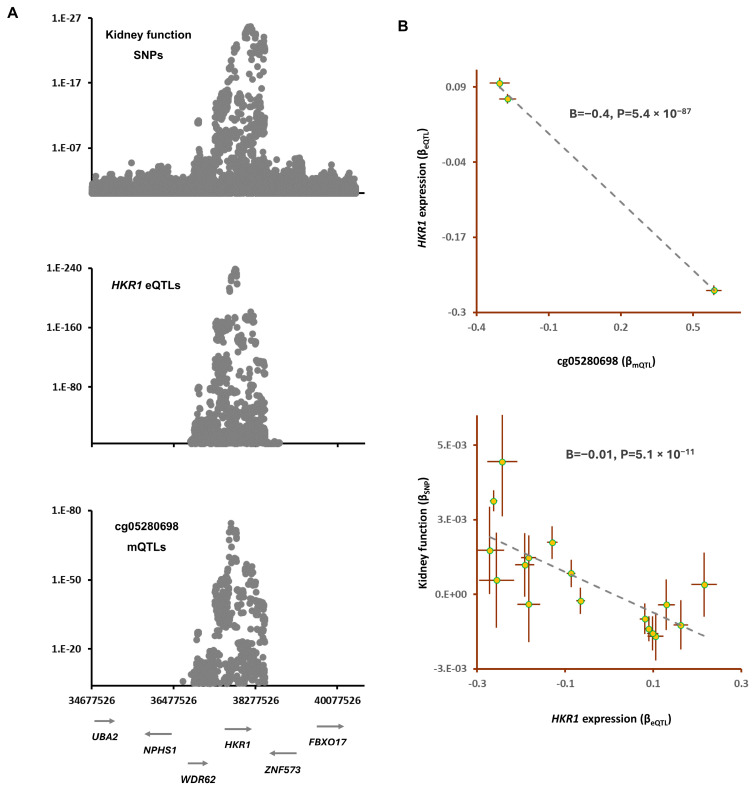
The mechanism whereby cg05280698 exerts the impact of vitamin B12 supplementation on kidney function: (**A**) regional association plots for mQTLs of cg05280698, eQTLs of *HKR1* and SNPs for kidney function overlap. The cg05280698 site is reported to become hypermethylated in people who take vitamin B12 supplementation (Table 3); and (**B**) MR analysis revealed that as the site becomes methylated, the expression of *HKR1* decreases and that this leads to higher kidney function. Complete statistical details are available in Table 4. Points on MR plots represent SNPs; the x-value of an SNP is its effect size on the predictor, and the horizontal error bar indicates the standard error around the effect size. Similarly, the y-value of the SNP indicates its effect size on the outcome, and the vertical error bar indicates the standard error. The dashed line represents the line of best fit (a line with the intercept of 0 and the slope of B from the MR test).

Yadava et al. reported [10] that vitamin B12 supplementation increases the methylation level at the cg05280698 site (Table 3). The colocalization analysis revealed that the mQTLs at this site overlap with a GWAS locus for kidney function (P_SMR_ = 4.0 × 10^−17^, P_HEIDI_ = 0.04, Table 1). The lead SNP in this region, rs320881(G), was associated with higher methylation at the cg05280698 site (B = 0.59, B = 2.1 × 10^−75^) and with higher kidney function (B = 0.003, *p* = 2.9 × 10^−21^). The outcome of the MR analysis also revealed that higher methylation at the cg05280698 site contributes to higher kidney function (B = 0.01, *p* = 2.3 × 10^−9^, Table 4). Finally, by integrating eQTL data, I identified *HKR1* as the gene that mediates the impact of the cg05280698 site on kidney function (Figure 4). It appears that, as the cg05280698 site becomes methylated, the expression of *HKR1* decreases (B = −0.42, B = 5.4 × 10^−87^) and this leads to higher kidney function (B = −0.01, B = 5.1 × 10^−11^, Figure 4). *HKR1* is a member of the Krüppel-like family of transcription factors, which are zinc finger DNA-binding proteins that regulate gene expression.

### 2.4. cg03186999-CTDNEP1-SBP

According to the data from the EWAS Atlas, air pollution lowers the methylation level at the cg03186999 site (Table 3); furthermore, the outcome of the SMR analysis indicated that the mQTLs of cg03186999 and SNPs for systolic blood pressure (SBP) colocalize (P_SMR_ = 4.6 × 10^−16^, P_HEIDI_ = 0.01; Table 1).

**Figure 5:**
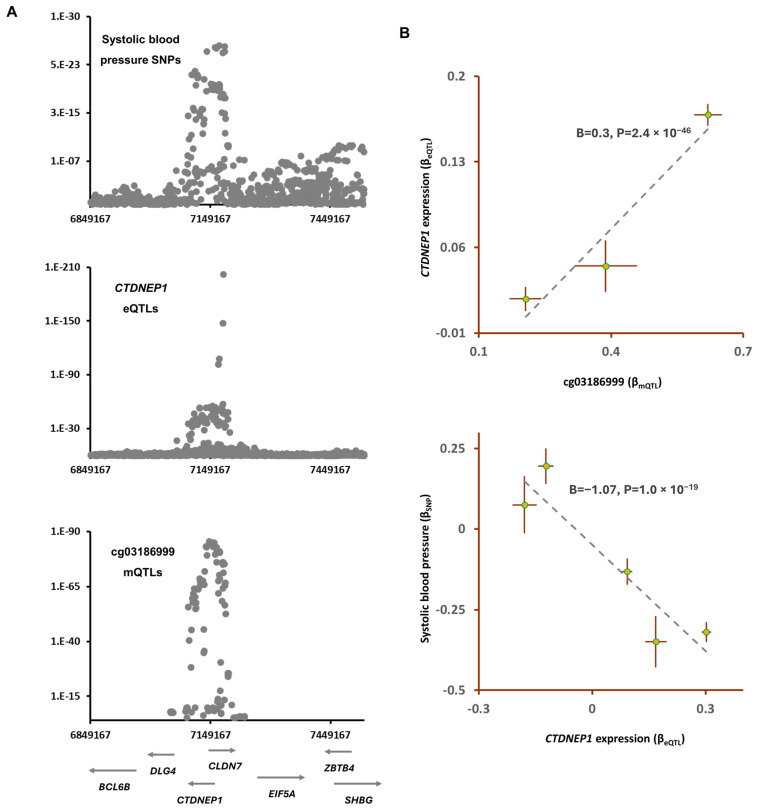
cg03186999 mediates the impact of air pollution on systolic blood pressure (SBP) by lowering the expression of *CTDNEP1*: (**A**) I noted an overlap between mQTLs of cg03186999, SNPs for SBP, and eQTLs for *CTDNEP1*. The cg03186999 site is reported to be hypomethylated in individuals exposed to air pollution (Table 3); and (**B**) the outcome of the MR analysis confirmed that as the cg03186999 site becomes hypomethylated, the expression of *CTDNEP1* decreases; this leads to higher SBP. Complete statistical details are available in Table 4. Points on MR plots represent SNPs; the x-value of an SNP is its effect size on the predictor, and the horizontal error bar indicates the standard error around the effect size. Similarly, the y-value of the SNP indicates its effect size on the outcome, and the vertical error bar indicates the standard error. The dashed line represents the line of best fit (a line with the intercept of 0 and the slope of B from the MR test).

The lead SNP in this region, rs402514(T), was associated with lower SBP (B = −0.28, *p* = 5.1 × 10^−19^) but higher methylation at the cg03186999 site (B = 0.62, *p* = 8.0 × 10^−86^). The outcome of Mendelian randomization further confirmed that lower methylation at this site contributes to higher SBP (B = −0.4, *p* = 7.2 × 10^−16^; Table 4). Furthermore, by integrating eQTL data, I found that *CTDNEP1* is the gene that mediates the impact of cg03186999 sites on SBP. The outcome of the MR analysis revealed that higher methylation at cg03186999 contributes to higher expression of *CTDNEP1* (B = 0.3, *p* = 2.4 × 10^−46^, Figure 5) and that this consequently lowers the systolic blood pressure (B = −1.07, *p* = 1.0 × 10^−19^). *CTDNEP1* encodes a phosphatase enzyme that is known to be involved in various biological processes.

## 3. Discussion

This study summarizes the outcomes of analyses in which, by integrating several publicly available datasets, molecular paths through which environmental chemicals influence cardiometabolic traits were investigated. Through a discovery and replication design, and by applying rigorous statistical criteria, four CpG sites and their related genes were identified that convey the impacts of environmental factors on cardiometabolic traits. The identified CpG sites could be tracked to assess the progress of a disease in individuals who are exposed to a chemical agent. Furthermore, given that the detected CpG–trait associations indicate causality (due to the nature of the Mendelian randomization test), the CpG sites could be targeted by epigenome editing approaches, such as CRISPRoff [11], for therapeutic interventions. It is notable that epigenomic changes are gradually reversible in response to external factors. As such, in situations where CRISPRoff is not possible, lifestyle modification is an alternative therapeutic remedy. The identified genes provide insight into the mechanisms through which a chemical substance impacts a trait. In the following paragraphs, their functions and relevance with respect to the identified traits are discussed.

In this study, the cg23627948 site was identified as mediating the impact of organophosphate [8] and maternal lead exposure [9] on obesity through the *GNA12* gene, which is a member of the G protein-coupled receptor α family. Previous studies underlined the role of *GNA12* in adipogenesis and energy expenditure [12,13,14]. It was reported that *GNA12*-encoded protein stimulates the proliferation, and inhibits the differentiation, of preadipocytes [14]. Furthermore, GNA12 facilitates whole-body energy expenditure through USP22/SIRT1-regulated mitochondrial respiration [13]. GNA12 levels were also shown to be lower in the liver of high-fat-diet-fed mice and in patients with steatosis and/or nonalcoholic steatohepatitis [13]. These findings, as well as the involvement of *GNA12* in different physiological processes, suggest that the contribution of this gene to obesity could be through different paths.

In the chromosome region 15q51.1, two genes, *CHP1* and *GCHFR*, were identified that mediated the impact of higher methylation at the cg21153102 site on diastolic blood pressure. The site is reported to become methylated as a result of exposure to prenatal perfluorooctane sulfonate [15]. *CHP1*, also known as calcineurin-like EF-hand protein 1, encodes a protein that is involved in various cellular processes. It acts as an endogenous inhibitor of calcineurin activity and thus may lead to hypertension through this path, given that a side effect of immunosuppressive medications that act as calcineurin inhibitors is hypertension [16]; moreover, CHP1 serves as an essential cofactor that supports the physiological activity of NHE family members, which are transmembrane proteins that act as a sodium–hydrogen antiporter. NHE proteins are important in regulating intracellular pH and in maintaining blood pressure homeostasis [17]. The influence of *GCHFR* on blood pressure could be attributed to its role in the production of the vasodilator molecule, nitric oxide. GCHFR has a regulatory role in the synthesis of BH4 (tetrahydrobiopterin) in endothelial cells, acting as an essential cofactor in the production of nitric oxide [18].

*HKR1* is a member of the Krüppel-like family of transcription factors, which are zinc finger DNA-binding proteins that regulate gene expression. In this study, I found a methylation site within this gene that, as it becomes methylated, increases the expression of *HKR1*; this also contributes to higher kidney function. The site is reported to be methylated in people taking B12 supplements [10]. It is of note that the site is also reported to become methylated as a result of exercise [19]. The role of the *HKR1* gene in kidney function remains unknown; however, in a recently published study, Liu et al. [20] conducted a comprehensive investigation of the molecular biology of kidney function in humans, they identified kidney-specific genes and catalogued methylation sites that impacted the function of such genes. Among their findings, they documented that the methylation of the *HKR1* gene changed the expression of this gene with regard to kidney function.

*CTDNEP1* is another gene associated with blood pressure. It mediate the impact of air pollution→cg03186999 site hypometylation [21] on systolic blood pressure. The CTDNEP1 protein, also known as C-terminal domain nuclear envelope phosphatase 1, is a member of the protein phosphatase family and has been recognized for its roles in various biological processes. Its contribution to blood pressure could be through its regulatory function in bone morphogenetic protein and the Wnt signaling pathway [22]. Furthermore, CTDNEP1 is known to dephosphorylate LPIN1, which is implicated in the development of hypertension [22,23]. Both CTDNEP1 and LPIN1 participate in lipid metabolism [24]. LPIN1-deficient mice were reported to have high systolic blood pressure [23]. Therefore, a path through which CTDNEP1 impacts blood pressure could be through lipid metabolism.

This study provides a framework for future studies that aim to investigate the molecular path through which an environmental factor impacts a trait. It shows, by connecting several disjointed data to the genome (i.e., SNPs), that it is possible to investigate their inter-dependencies and infer the underlying molecular mechanism. Nonetheless, it has several limitations that future studies can improve upon. In both the discovery and replication stages, mQTL data were obtained from Illumina HumanMethylation450K Beadchip, which covers about 1.6% (450,000 CpG sites) of the CpG sites in the human genome [25]. Therefore, conducting EWAS studies using more dense methylation arrays is necessary. Furthermore, considering that DNA methylation is just one form of epigenomic modification, cataloguing the SNPs underpinning other forms of epigenome modifications is important. Tissue specificity is another factor to consider. In this study, I used mQTL and eQTL data generated using blood samples; however, blood is an intermediary tissue. It is more appropriate to conduct the analyses using data from tissues that are pertinent to the trait of interest.

The findings of this study were obtained by examining data from studies conducted with European populations. This minimizes the likelihood of population stratification; however, it raises concerns with regard to the generalizability of the results. A recent study by Hatton et al. [26] indicates that the genetics of DNA methylation is largely shared across European and east Asian populations. Findings from studies that compare the genetics architecture of traits across ancestries indicate similar findings [27,28]. Therefore, the generalizability of the findings should not be a concern; transethnic studies could be performed to identify the underlying biomarkers with more molecular precision and higher statistical power.

As reviewed earlier [5], previous studies that catalogued epigenome sites associated with chemical exposure suffer from small sample sizes. As such, large cohorts and collaborative meta-analyses are required to comprehensively investigate the impact of environmental chemicals → epigenome modifications on disorders.

In this study, I used Mendelian randomization to investigate whether changes in methylation at a CpG site have causal impacts on the endpoint trait. MR fulfills this goal by comparing the pattern of association between the natural variants in the genome (i.e., SNPs) with the CpG site, as well as with the trait of interest. The determination of alleles of SNPs occurs during meiosis and it is a random process (i.e., unaffected by environmental factors). Therefore, any concurrent association that we identify between segments of the genome with the methylation level at a CpG site and a trait is a genuine association. One issue that might occur in this context is the weak instrument bias or the phenomenon that SNPs associated with the predictor collectively explain a small portion of the phenotypic variance of the predictor. This is especially correct when the predictor shows a polygenic mode of inheritance or is under the regulatory impact of many SNPs. However, in the current study, this is less of an issue because the examined predictors were CpG sites that, unlike a polygenic trait, are under the regulatory control of fewer SNPs and, as such, are less likely to suffer from weak instrument bias.

## 4. Conclusions

In summary, this study provides a list of CpG sites and their genes that mediate the impact of environmental chemicals on cardiometabolic traits. The CpG sites identified in this study could be monitored for early diagnosis. Furthermore, they could be targeted for therapeutic interventions through universal epigenome editing approaches such as CRISPRoff. The multiomics approach described in this article provides a convenient workflow that allows for the investigation of the impacts of environmental factors on biomarkers of the body, and, consequently, on health conditions, using publicly available data.

## 5. Materials and Methods

### 5.1. Data Sources

The EWAS Atlas [29] is a curated database of epigenome-wide association studies in which the authors categorized CpG sites according to the nature of their associations with traits into different categories such as cancer, behavior, phenotype, non-cancer disease, and environmental factors. From these categories, I initially selected CpG sites associated with environmental factors and further refined the list by excluding non-chemical factors. The resulting CpG sites were then examined using the procedure described in Figure 1 to investigate their impacts on cardiometabolic traits.

mQTLs underlying CpG sites were obtained from a study by McRae et al. [30], in which the authors used the Illumina HumanMethylation450 array to measure DNA methylation in blood samples taken from 1,980 subjects of European descent. GWAS data for cardiometabolic traits were also obtained from studies (Table 1) conducted on European populations to minimize the possible bias due to population stratification. Consequently, to compute the extent of linkage equilibrium (LD) between SNPs, I used genotype data from the European sample (n = 503) of the 1000 Genomes Project (phase 3).

mQTL data from the Aberdeen Genetics Study [31] were used for the replication step. In this study, the authors investigated mQTLs in blood samples taken from 847 individuals of British origin using the Illumina HumanMethylation450 array.

To investigate genes that mediated the impact of CpG sites on the traits, eQTL data from the eQTLGen consortium [32] were obtained and integrated into the analysis. The eQTLGen consortium represents a collaborative effort in which the authors investigated the genetic architecture of blood gene expression by incorporating eQTL data from 37 datasets, compromising a total of 31,684 individuals of primarily European ancestry.

### 5.2. Analyses

Initially, the relationship between a CpG site and a cardiometabolic trait was investigated by comparing their patterns of association with SNPs. From the statistical point of view, this is called a colocalization test; the aim of the test is to find loci where SNP association signals for a CpG site and a trait overlap. In this study, the colocalization test was conducted using SMR software (version 1.3.1) [6]; the underlying algorithm searches for a colocalization pattern by comparing the association of the lead SNP (most significantly associated SNP) within a locus with both the CpG site and the trait, and then evaluating the impact that the SNP exerts on the trait through the CpG site. In this context, a significant association (P_SMR_ < 0.05) could imply pleiotropy (SNP has a regulatory impact on both the CpG site and the trait) or linkage (the actual causal SNPs are different, and the lead SNP is merely in LD with them). To rule out the possibility of a linkage effect, SMR uses a statistical test known as heterogeneity in dependent instruments (HEIDI). In summary, the test can identify a linkage effect (P_HEIDI_ ≤ 0.01) by comparing the association of SNPs surrounding the lead SNP with both the CpG site and the trait. In this context, if a heterogeneity is observed in the pattern of associations, it indicates linkage. Following this step, CpG site–trait pairs that their underlying SNPs colocalized (P_SMR_ < 5 × 10^−8^, P_HEIDI_ > 0.01) were then re-examined through the replication analysis to investigate the possibility of spurious associations. Inherently, SMR cannot test whether a change in the level of methylation at a CpG site has a causal impact on a trait because in order to make a causal inference, multiple independent SNPs are required. As such, Mendelian randomization (MR) [7] was then used to inspect the findings from the colocalization step and identify CpG sites having causal impacts on traits.

Mendelian randomization (MR) is a form of instrumental variable analysis that investigates the relation between the predictor (CpG site) and the outcome (trait) using an instrument (a set of independent SNPs) known to cause changes in the predictor. The test works by drawing the impact of SNPs on a CpG site and its corresponding trait on a scatterplot and calculating the slope (B) of the fitted line and the variance (SE) around it. In this context, a significant positive B indicates that subjects genetically susceptible to higher methylation at the CpG site tend to have higher trait values.

SNPs selected for the MR test must possess the following criteria: (a) they must not be in linkage disequilibrium; (b) they must be significantly associated with the CpG site; and (c) they must not show a pleiotropic effect (i.e., CpG site ← SNP → Trait). For the purpose of this study, the degree of linkage disequilibrium between SNPs was set at r^2^ ≤ 0.05 and the degree of association between an SNP and its CpG site was set at *p* ≤ 5 × 10^−8^; moreover, SNPs that showed a pleiotropic effect were excluded using the HEIDI test (P_HEID_ > 0.01). The benefit of using an instrument with SNPs to investigate the relationship between two entities is that such an instrument is inherently immune to the confounding effect of environmental factors that can bias an association test. This is because the alleles of independent SNPs are allocated to offspring at conception (Mendel’s second law) through a random process (i.e., unaffected by environmental factors). It is notable that, considering that pleiotropic SNPs are excluded from the MR test, the findings do not indicate correlation (CpG site ↔ Trait); furthermore, by swapping the places of the predictor and the outcome and repeating the test, MR analysis enables the investigation of the possibility of reverse causation (Trait → CpG site). In this study, I used the GSMR algorithm (version 1.1.1) [7] to conduct MR analysis. GSMR has several helpful functions that facilitate the analyses such as filtering out the pleiotropic SNPs, aligning the effect alleles of the predictor and the outcome to the same reference allele, as well as taking the linkage disequilibrium between SNPs and variances around effect sizes into account.

CpG site–traits that showed a significant association following MR analysis (forward MR *p* < 5 × 10^−8^ and reverse MR *p* > 0.05) were then subjected to functional investigation by integrating eQTLs from the eQTLGen consortium. The purpose of this step was to identify genes that mediate the impact of CpG sites on traits. The analysis was initiated by first identifying CpG–gene pairs that share significant associations with an SNP (*p* < 5 × 10^−8^). Next, MR analysis was used to test if changes in the methylation level at the CpG site have a causal impact on expression of the gene. If a significant association was detected (*p* < 5 × 10^−8^), the analysis was then extended by examining the association between the gene and the target trait. Following this step, functional information from various web resources (including Microsoft Copilot AI) were studied to infer the possible link between a gene and its trait.

## Figures and Tables

**Table 1:** Colocalization analysis revealed CpG sites that their mQTLs overlap with the SNPs of cardiometabolic traits.

Trait (Source)	CpG Site	Lead SNP (A1 Allele)	Association *		Colocalization Results		
			B	*p*	B	P_SMR_	P_HEIDI_
Body fat percentage (UKBB)	cg23627948	rs798549(C)	0.01	1.3 × 10^−8^	0.01	1.4 × 10^−8^	0.04
			1.41	<2 × 10^−200^			
DBP (UKBB)	cg21153102	rs4924526(A)	0.17	2.5 × 10^−23^	0.18	8.0 × 10^−22^	0.3
			0.99	<2 × 10^−200^			
Kidney function (PMID: 31152163)	cg05280698	rs320881(G)	0.003	2.9 × 10^−21^	0.01	4.0 × 10^−17^	0.04
			0.59	2.1 × 10^−75^			
SBP (UKBB)	cg03186999	rs402514(T)	−0.28	5.1 × 10^−19^	−0.45	4.6 × 10^−16^	0.01
			0.62	8.0 × 10^−86^			

* indicates the association of the lead SNP with the trait (first row) and the CpG site (second row).

**Table 2:** Colocalization of mQTLs of CpG sites and SNPs of cardiometabolic traits in the replication study.

Trait (Source)	CpG Site	Lead SNP (A1 Allele)	Association *		Colocalization Results		
			B	*p*	B	P_SMR_	P_HEIDI_
Body fat percentage (UKBB)	cg23627948	rs798549(C)	0.01	1.3 × 10^−8^	0.06	1.61 × 10^−8^	0.3
			0.15	<2 × 10^−200^			
DBP (UKBB)	cg21153102	rs11070317(C)	0.18	1.7 × 10^−24^	2.06	6.1 × 10^−23^	0.3
			0.09	5.2 × 10^−294^			
Kidney function (PMID: 31152163)	cg05280698	rs73025481(A)	0.004	2.3 × 10^−23^	0.04	2.5 × 10^−16^	0.02
			0.08	3.2 × 10^−47^			
SBP (UKBB)	cg03186999	rs222851(A)	−0.27	8.6 × 10^−19^	−11.22	4.3 × 10^−14^	0.03
			0.02	1.7 × 10^−47^			

* indicates the association of the lead SNP with the trait (first row) and the CpG site (second row).

**Table 3:** Association of CpG sites identified in this study with environmental chemicals, according to the EWAS Atlas data.

Trait	CpG Site	Correlation	Sample Size	*p*-Value	PMID
Prenatal lead exposure	cg23627948	−	268	7.8 × 10^−5^	28858830
Organophosphate exposure	cg23627948	+	580	2.2 × 10^−7^	30248838
Prenatal perfluorooctane sulfonate (PFOS) exposure	cg21153102	+	266	1.0 × 10^−5^	35266797
Vitamin B12 supplement	cg05280698	+	12	5.0 × 10^−7^	29135286
Air pollution (Pb)	cg03186999	−	695	2.0 × 10^−10^	34717175
Air pollution (Na)	cg03186999	−	695	2.8 × 10^−13^	34717175

**Table 4:** The outcome of Mendelian randomization; nature of association between the identified CpG sites, genes, and cardiometabolic traits.

Predictor	Outcome	B	SE	*p*	N_SNPs_
cg23627948 → GNA12 → Obesity					
cg23627948	Body fat percentage	0.01	0.001	1.0 × 10^−8^	17
cg23627948	GNA12	−0.10	0.007	4.4 × 10^−47^	7
GNA12	Body fat percentage	−0.03	0.004	4.5 × 10^−12^	20
cg21153102 → GCHFR/CHP1 → DBP					
cg21153102	DBP	0.18	0.02	1.8 × 10^−23^	12
cg21153102	CHP1	−0.15	0.009	1.7 × 10^−53^	12
cg21153102	GCHFR	0.05	0.008	1.9 × 10^−11^	7
CHP1	DBP	−0.57	0.08	9.8 × 10^−13^	6
GCHFR	DBP	0.39	0.06	4.1 × 10^−10^	9
cg05280698 → HKR1 → Kidney function					
cg05280698	Kidney Function	0.01	0.001	2.3 × 10^−9^	3
cg05280698	HKR1	−0.42	0.02	5.4 × 10^−87^	3
HKR1	Kidney Function	−0.01	0.001	5.1 × 10^−11^	17
cg03186999 → CTDNEP1 → SBP					
cg03186999	SBP	−0.44	0.05	7.2 × 10^−16^	3
cg03186999	CTDNEP1	0.26	0.02	2.4 × 10^−46^	3
CTDNEP1	SBP	−1.05	0.1	1.0 × 10^−19^	5

## Data Availability

The list of CpG sites that undergo modification in response to environmental chemicals was obtained from the EWAS Atlas database: https://ngdc.cncb.ac.cn/ewas/atlas (accessed on 26 July 2024). mQTL summary statistics were obtained from: https://yanglab.westlake.edu.cn/software/smr/#DataResource (accessed on 26 July 2024). eQTL summary statistics were obtained from the eQTLGen consortium: https://www.eqtlgen.org/ (accessed on 26 July 2024). GWAS summary statistics for cardiometabolic traits were obtained from UK Biobank: https://www.ukbiobank.ac.uk/ (accessed on 26 July 2024) and CDKGen consortium: https://ckdgen.imbi.uni-freiburg.de/ (accessed on 26 July 2024).
